# Radiation-induced cavernous malformation in the brainstem after Gamma Knife radiosurgery for vestibular schwannoma: A case report and literature review

**DOI:** 10.1016/j.bas.2026.105999

**Published:** 2026-03-02

**Authors:** Erlend Moen Taule, Henrik Broch Kvernaas, Tor-Christian Aase Johannessen, Tormund Haugland Njølstad, Øystein Vesterli Tveiten, Rupavathana Mahesparan, Terje Sundstrøm

**Affiliations:** a Department of Neurosurgery, Haukeland University Hospital, Bergen, Norway; b Akershus University Hospital, Lørenskog, Norway; c Department of Oncology, Haukeland University Hospital, Bergen, Norway; d Department of Radiology, Haukeland University Hospital, Bergen, Norway; e Department of Clinical Medicine, University of Bergen, Norway

**Keywords:** Cavernous malformation, Vestibular schwannoma, Radiotherapy, Gamma knife, Stereotactic radiosurgery

## Abstract

**Introduction:**

Radiation-induced cavernous malformation (RICM) is an uncommon late complication of radiation therapy. There are even fewer cases reported after stereotactic radiosurgery (SRS). In this study, we investigated the clinical characteristics, management considerations, and outcomes of RICM following SRS.

**Research question:**

What are the clinical characteristics, management considerations, and outcomes of RICM following SRS?

**Material and methods:**

We describe a case of a 50-year-old woman previously treated with Gamma Knife® radiosurgery for a vestibular schwannoma. Almost two decades later, brain magnetic resonance imaging revealed a brainstem cavernous malformation in the brainstem, in the dose fall-off region adjacent to the target volume. A literature review was subsequently conducted to identify comparable cases, associated therapeutic strategies, and clinical outcomes.

**Results:**

We identified 32 reported cases of RICM following SRS in the literature. The mean age at SRS was 65 years, with 57% being female. Mean latency to RICM development was 7.3 years. Our case demonstrated one of the longest latency periods reported for this complication. The patient had mild symptoms and was managed conservatively with surveillance imaging. Literature review revealed that 78% of cases underwent surgical management, predominantly those presenting with hemorrhage or progressive neurological symptoms.

**Conclusion:**

RICM represents a rare but clinically significant late complication of SRS that can occur after extended latency periods, even in adults. Management should be individualized based on symptoms, hemorrhage history, and lesion location. Further research is needed to develop more evidence-based management of RICM, and to better define the true incidence through long-term follow-up studies.

## Highlights

• Radiation-induced cavernous malformations (RICMs) have historically been associated with conventional fractionated radiotherapy or whole brain radiation in children.
• 32 cases of RICMs following stereotactic radiosurgery were identified in our literature review, and patients were treated for a range of different conditions.
• We describe a brainstem RICM presenting 19 years after Gamma Knife® radiosurgery for vestibular schwannoma, emphasizing the potential for very long latency intervals.
• RICMs after stereotactic radiosurgery are poorly documented in the literature.

## 1 Introduction

Cavernous malformations are benign vascular lesions located in the central nervous system, with an estimated prevalence of approximately 0.5% in the general population (Rigamonti et al., 1988; Wong et al., 2000). Radiation-induced cavernous malformations (RICMs) represent a rare sequela of cranial radiation and are most frequently observed in individuals treated during childhood (Patet et al., 2022). Although RICMs have been reported following radiation therapy for a variety of neoplastic conditions, they are usually associated with pediatric malignancies such as medulloblastoma and leukemia (Koester et al., 2023; Nimjee et al., 2006). Since Wilson's first description in 1992 (Wilson, 1992), over 300 cases of RICMs have been documented across different radiation modalities (Koester et al., 2023; Vacek and Kaliaperumal, 2024), with only four cases reported after stereotactic radiation for vestibular schwannoma (Graboyes et al., 2025; Nussbaum et al., 2019; Sasagawa et al., 2009; Murakami et al., 2011).

In this report, we describe a case of a brainstem RICM occurring nearly two decades after Gamma Knife® radiosurgery (GKRS) for a vestibular schwannoma. We also provide a narrative review of the literature, focusing specifically on RICMs arising after SRS.

## 2 Case report

**Fig. 1:**
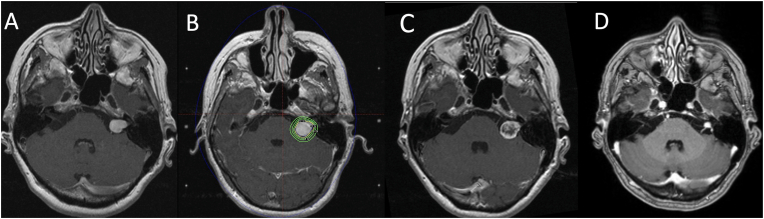
**Radiological course and treatment of the vestibular schwannoma**. Temporal progression from initial tumor diagnosis in 2003 to the most recent follow-up assessment of tumor control in 2019 on axial contrast-enhanced T1 weighted MR images. A) MRI obtained at the time of diagnosis in 2003 demonstrates an avidly enhancing lesion in the left cerebellopontine angle extending into the internal auditory canal, consistent with a Koos grade II vestibular schwannoma. B) MRI acquired for Gamma Knife® radiosurgery (GKRS) planning and dose plan in 2005 following tumor growth, with a cisternal component indenting the left cerebellar peduncle. C) Follow-up imaging in 2006 demonstrates central loss of enhancement, consistent with treatment-related necrosis. D) MR images obtained 14 years after GKRS shows a shrunken, avidly enhancing vestibular schwannoma in the left cerebellopontine angle.

A 50-year-old woman initially underwent cranial computed tomographic (CT) imaging in 2002 due to progressive left-sided hearing loss and a sensation of aural fullness. Subsequent magnetic resonance imaging (MRI) in 2003 revealed a 16 mm contrast-enhancing lesion within the left internal auditory canal with a small cisternal component, consistent with a vestibular schwannoma Koos grade II (Fig. 1A). The patient was managed conservatively with regular clinical and radiological surveillance.

By 2005, her symptoms had progressed to include tinnitus and dizziness, and imaging showed tumor enlargement to 19 mm with a cisternal component indenting the brainstem. She subsequently underwent GKRS, with a target tumor volume of 2 cm^3^ using a 30% isodose of 12 Gy to the periphery (Fig. 1B). The 12 Gy-volume was 2.268 cm^3^ and the 6 Gy-volume was 6.481 cm^3^. The brainstem and cerebellar peduncle area where she later developed a cavernous malformation, received a fall-off dose range of 3-10 Gy. Post-treatment follow-up demonstrated a favorable response, with central necrosis (Fig. 1C) and gradual involution of the tumor to a diameter of 7 mm by 2015. A follow-up MRI in 2019, 14 years after radiosurgery, showed a stable, shrunken lesion (Fig. 1D), and routine surveillance was discontinued.

**Fig. 2:**
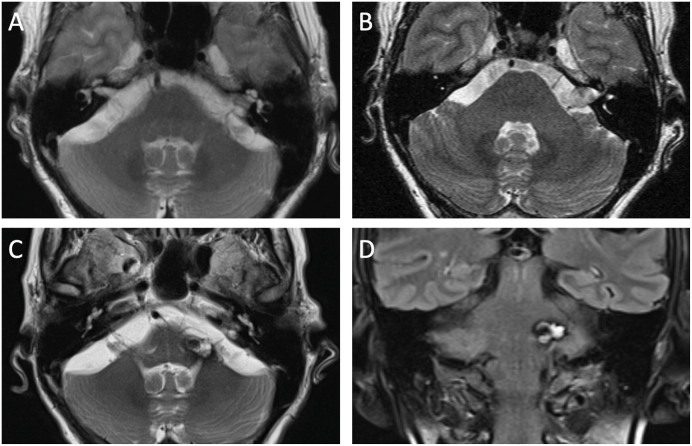
**Radiation-induced cavernous malformation formation**. Selection of MR images demonstrating time course of development of RICM. A) Axial T2-weigthed MR image in 2004, prior to Gamma Knife® radiosurgery (GKRS) of left vestibular schwannoma. B) Follow-up axial T2-weigthed MR image in 2017, 12 years after radiosurgery, with no evidence of a cavernous malformation. C) Axial T2-weigthed and D) coronal Fluid-Attenuated Inversion Recovery (FLAIR) MR images in 2024, 19 years after GKRS, shows a lobulated hemorrhagic lesion with mixed signal intensity and hemosiderin ring in the left cerebellar peduncle, suggestive of a cavernous malformation.

In 2024, the patient presented to the emergency department following a pertrochanteric femoral fracture. Clinical evaluation revealed a history of increased falls over the preceding weeks. A brain MRI was obtained as part of the workup and revealed a lesion in the left cerebellar peduncle with central areas of lobulated high T2-signal and a low-signal hemosiderin ring, radiologically consistent with a cavernous malformation (Fig. 2C and D). Previous T2-weighted images did not show any brainstem pathology in the same area (Fig. 2A and B). Given the patient's age, the lesion's location in eloquent brainstem tissue, the asymptomatic nature of the finding, and the relatively low risk of hemorrhage associated with observation, a conservative management approach was adopted. The patient was counseled regarding the risks and benefits of observation versus surgical intervention and elected for conservative management.

## 3 Discussion

RICMs are a well-known occurrence after whole brain radiation (Patet et al., 2022; Nimjee et al., 2006). However, they have only been reported a few times after SRS (Nussbaum et al., 2019; Wang et al., 2018) and was first described by Pozatti et al., in 1996 (Pozzati et al., 1996). The incidence of RICMs following SRS has been estimated at approximately 0.9% at 15 years of follow-up (Nagy et al., 2018). Although considered uncommon, Kim et al. identified RICMs in 3 of 20 patients (18.6%) treated with GKRS for mesial temporal lobe epilepsy over an 8-year follow-up period when they systematically screened for their presence (Kim et al., 2024). A narrative literature search identified 32 reported cases of RICM following SRS (Table 1). The mean age at the time of SRS was 65 years (range: [12-78] years), with a slight female predominance (57%, 16/28 cases with documented gender). The primary indications for SRS included brain metastases (n = 6), meningiomas (n = 6), arteriovenous malformations (n = 4), vestibular schwannomas (n = 4), a trigeminal schwannoma (n = 1), gliomas (n = 3), epilepsy (n = 4), cavernous malformations (n = 2), and a pineocytoma (n = 1). The mean latency period from SRS to RICM diagnosis was 7.3 years (range: [0.7-21] years), with our case representing one of the longest reported intervals at 19 years.

**Table 1:** Summary of reported cases of radiation-induced cavernous malformation following stereotactic radiosurgery.

	Age (y), sex	Dx prompting prior radiation	Location Dx	Type of radiation	Latency	Location RICM	Presenting symptoms	Treatment
Pozzati et al. (1996)	13, W	CM	FL	GKRS (20-50 Gy)	10 y	Supra- and infratentorially (multiple)	Seizures	Surgery (right frontal lobe)
Iwai et al. (2007)	37, M	BM	TL	GKRS x 2 (23 Gy + 23 Gy)	2 y	TL	Headache, seizure, diplopia, oculomotor nerve palsy.	Surgery
Motegi et al. (2008)	47, M	AVM	CN	Linear accelerator (25 Gy)	6.5 y	CN	Generalized seizure	Conservative, later surgery
Sasagawa et al. (2009)	35, M	VS	CPA	GKRS (12 Gy)	10 y	Pons	Hemihyperesthsia and mild hemiparesis	Conservative
Yeon et al. (2010)	33, W	CM	LN	GKRS (13 Gy)	3.5 y	CN	Headache, nausea and vomiting	Surgery
Park et al. (2011)	21, W	Pineocytoma	Epithalamus	GKRS (14.5 Gy)	3 y	Brainstem	Facial palsy and mental status changes	GKRS
Murakami et al. (2011)	39, W	VS	CPA	GKRS (11.3)	14 y	CPA	Vestibulocochlear symptoms	Surgery
Murakami et al. (2011)	33, W	TS	Middle cranial fossa	GKRS (12 Gy)	2 y	Middle cranial fossa/prepontine cistern	Trigeminal and abducens symptoms, and contralateral sensorial symptoms	Surgery
Wang et al. (2012)	59, M	AVM	Cerebellum	GKRS (20 Gy)	6 y	Cerebellum	Gait unsteadiness and headache	Surgery
Kunitaka et al. (2018)	12, M	PA	Suprasellar	cRT (60 Gy, 2 y before SRS) + Cyberknife (33.6 Gy)	4 y	Thalamus	Left hemiparesis	Medically
Nagy et al. (2018)	75, NR	Meningioma	TL	GKRS (13 Gy)	2 y	TL	Asymptomatic	Observation
Nagy et al. (2018)	45, NR	Meningioma	Pons	GKRS (15 Gy)	10 y	Pons	ICH	Observation
Nagy et al. (2018)	47, NR	Meningioma	TL	GKRS (18 Gy)	21 y	TL	Asymptomatic	Observation
Nagy et al. (2018)	46, NR	Meningioma	OL	GKRS (NR)	7 y	OL	Seizure, cognitive decline, visual loss	Bevacizumab, surgery
Wang et al. (2018)	45, M	Glioma	Vermis	GKRS (14 Gy)	2 y	Vermis	Gait disturbance and severe headache	Conservative for 2 y, then surgery
Winkler et al. (2018)	33, W	Epilepsy	TL	GKRS (20 Gy)	9.5 y	TL	Multiple falls, complex partial seizures and later limited interaction and lethargy before loss of consciousness and several neurological findings	First conservative, later surgery
Burkhardt et al. (2019)	70∗, M	AVM	TL	Radiosurgery (NR)	NR	TL	Aphasia and confusion	Surgery
Nussbaum et al. (2019)	42, W	VS	CPA	Cyberknife linear accelerator SRS (25 Gy)	11 y	CPA	Facial numbness	Surgery
Seiger et al. (2019)	20, W	BM	FL, cerebellum	WBRT (30 Gy, 1.4 y before SRS) + Cyberknife robotic linear accelerator (24 Gy x 3)	4.4 y post WBRT, 2.9 y post SRS	FL	NR	Surgery
Seiger et al. (2019)	25, W	BM	FL	MLC (multi-leaf collimator)-based linear accelerator SRS (20 Gy)	2 y	FL	NR	Surgery
Oishi et al. (2020)	50, W	Glioma	Left insular, invading the frontal and temporal lobes	CyberKnife (35 Gy x 5)	9 y	TL	Speech disturbance	Conservative, then surgery
Oishi et al. (2020)	53, M	AE	OL	cRT (60 Gy, 1 y before SRS) + GKRS (20 Gy)	15 y	OL	Intermittent visual hallucinations	Surgery
Yu et al. (2020)	57, W	Meningioma	Right parasellar, cavernous sinus	SRS (12 Gy)	3 y	TL	Right eyelid droop, headaches and dizziness	Surgery
Takamori et al. (2020)	43, M	BM	Right lobe	SRS (30 Gy x 3)	8 months	Right lobe	Epileptic seizure	Surgery
Lee et al. (2022)	59, W	Meningioma	Sphenoid ridge	GKRS x 2 (13 + 15 Gy)	9,5 y and 12.5 y	Sphenoid ridge	Headache, aphasia, progressive cognitive decline.	Surgery
Chew et al. (2022)	67, W	BM	Multiple, largest in left inferior frontal lobe	GKRS (18.5-19.5, 4 metastasis)	30 months and 87 months	FL	Asymptomatic first time. Word finding and handwriting difficulties	Surgery two times
Patterson et al. (2022)	63, W	AVM	TL	SRS (20 Gy)	7 y	TL	Progressive confusion and expressive aphasia	Surgery
Kim et al. (2024)	29, M	MTLE	TL	GKRS (24 Gy)	7 y	TL	Headache and dizziness	Surgery
Kim et al. (2024)	16, M	MTLE	TL	GKRS (24 Gy)	7 y	TL	Progressive visual disturbance	Surgery
Kim et al. (2024)	32, M	MTLE	TL	GKRS (24 Gy)	7 y	TL	Headache	Conservative
Hajikarimloo et al. (2025)	70s, W	BM	Missing	GKRS (missing)	Missing	PL	Missing	Surgery
Graboyes et al. (2025)	78, W	VS	CPA	Fractionated SRT (NR)	16 y	CPA	Hemifacial spasm	Surgery

AE: Anaplastic ependymoma, AVM: Arteriovenous malformation, BM: Brain metastasis, CM Cavernous malformation, cRT: Conventional radiotherapy, CN: Cranial nerve, CPA: Cerebellopontine angle, Dx Diagnosis, FL: Frontal lobe, GKRS Gamma Knife surgery, Gy: Grays, ICH: Intracerebral hemorrhage, LN: Lentiforme nucleus, M: Male, MTLE Mesial temporal lobe epilepsy, NR: Not reported, OL: Occipital lobe, PA: Pilocystic astrocytoma, PL: Parietal lobe, RICM: Radiation-induced cavernous malformation, SRS: Stereotactic radiosurgery, SRT: Stereotactic radiotherapy, TL: Temporal lobe, TS: Trigeminal schwannoma, VS: Vestibular schwannoma, W: Woman, WBRT: Whole-brain radiotherapy, y: years.

∗ age at date of operation

Previous reviews have demonstrated that RICMs are frequently asymptomatic (58% - 67%) and often discovered incidentally (Patet et al., 2022; Koester et al., 2023; Nussbaum et al., 2019). When symptomatic, the most common clinical manifestations include seizures, headaches, and focal neurological deficits. In a systematic review by Koester et al. they reported hemorrhage as the presenting feature in 13 of 21 patients (61%) who had undergone SRS, nearly double the rate observed after fractionated radiation, suggesting a higher likelihood of symptomatic presentation after stereotactic treatment (Koester et al., 2023). Similarly, Cutsforth-Gregory et al. found that RICMs exhibited a higher annual hemorrhage rate compared with non-radiation-induced cavernous malformations (4.2% vs 0.35%, respectively), although this difference did not reach statistical significance (Cutsforth-Gregory et al., 2015). This trend has been hypothesized to reflect greater vascular injury associated with single-session high-dose irradiation (Park et al., 2012).

The biological mechanisms underlying the development of RICMs remain incompletely understood. The prevailing hypothesis suggests that delayed vasculopathy characterized by hyalinization, fibrinoid necrosis of endothelial walls, and vascular proliferation plays a central role in their pathogenesis (Reinhold and Hopewell, 1980; Hassler and Movin, 1966; Heckl et al., 2002; Valk and Dillon, 1991; Poussaint et al., 1995). The extended latency periods observed in many cases, support a chronic, progressive pathological process rather than acute radiation injury. A dose-response relationship is biologically plausible, as radiation-induced vascular injury increases with dose. However, RICM is an extremely rare late complication, and the available evidence consist almost entirely of heterogenous case reports with limited dosimetric detail (Mariniello et al., 2019), making it difficult to define any clear tolerance threshold. In addition, individual patient factors likely influence risk, as differences in vascular vulnerability may explain why cavernoma develop in some patients but not in others despite similar radiation exposure. An important controversy exists regarding whether RICMs represent true cavernous malformations or radiation-induced vascular lesions that mimic cavernomas histologically. Some authors have raised concerns that the histology of RICMs may be pathologically distinct from non-radiation-induced cavernomas (Kim et al., 2024; Park et al., 2012; Reinhold and Hopewell, 1980). Thus, it has been argued that the term “pseudocavernoma” or “radiation-induced vascular malformation” might be more accurate for these lesions (Karlsson et al., 2019).

In the largest systematic review to date, Koester et al. reviewed 248 with RICMs from all types of radiation (Koester et al., 2023). Only 0.8% of the cases occurred following radiation therapy for vestibular schwannoma. Sasagawa et al. described the first reported case in 2009: A 35-year-old man previously treated with tumor resection and subsequent GKRS for a large vestibular schwannoma presented a decade later with hemihypesthesia and mild hemiparesis (Sasagawa et al., 2009). MRI findings showed a heterogeneous, “popcorn-like” lesion on T2-weighted imaging and a hyperintense core on T1-weighted imaging in the pons consistent with a cavernous malformation. The patient's symptoms improved with conservative management within ten days.

In 2011, Murakami et al. (2011) reported on a 39-year-old woman who 167 months after GKRS for a vestibular schwannoma was reoperated with pathological findings of a cavernous angioma. More recently, Nussbaum et al. (2019) reported a de novo cavernous malformation within the superior pole of a vestibular schwannoma 11 years after linear accelerator-based stereotactic radiosurgery. Surgical resection revealed a vascular lesion composed of irregular channels with variably hyalinized interfaces, macrophage infiltration, and collagen deposition, features more suggestive of a coagulum-like malformation rather than a true cavernoma, given the absence of back-to-back caverns and lack of SMA immunopositivity (Nussbaum et al., 2019; Kleinschmidt-DeMasters and Lillehei, 2016). Recently, Graboyes et al. reported a cavernous malformation that presented with hemifacial spasms 16 years after the patient was treated for a vestibular schwannoma with fractionated stereotactic radiotherapy. The lesion was operated, and trichrome staining showed hyalinized vessels and absence of muscular walls (Graboyes et al., 2025).

Management options for cavernous malformations include observation, surgical intervention, and, paradoxically in the context of RICM, stereotactic radiosurgery. According to an international consensus statement, treatment decisions should be guided by patient age, symptomatology, hemorrhagic history, and the lesion's location and size (Tasiou et al., 2023). A single-institution retrospective review by Koester et al. identified 10 patients with RICM; nine were managed surgically, with symptom improvement in five, worsening in three, and stability in one over a median follow-up of 15.5 months. The sole asymptomatic patient underwent observation. Among three patients treated for brainstem lesions, symptoms improved for two patients and worsened for one patient, highlighting both the potential benefits and risks of surgical intervention in this challenging location (Koester et al., 2023). Recent practice guidelines from the International Stereotactic Radiosurgery Society (ISRS) concluded, based on meta-analytic evidence, that SRS is an effective treatment for cavernous malformations, significantly reducing hemorrhage rates and improving seizure control in approximately 80% of cases, with radiographic response in 46.9% of cases (Tos et al., 2024). However, these guidelines did not specifically address RICMs, and the appropriateness of treating a radiation-induced lesion with additional radiation remains controversial. Park et al. (2011) reported the first case of a RICM treated with radiosurgery. The area where the lesion developed had previously received 5.0 ± 3.7 Gy during initial treatment and was subsequently managed with a margin dose of 12.5 Gy. Five years later, the malformation had reduced in size with no evidence of new hemorrhage, suggesting that repeat SRS may be a viable option in selected cases. Among published cases of RICMs arising after radiation treatment for vestibular schwannoma, three have been treated surgically and one managed conservatively (Table 2). In Nimjee et al. (2006) review of RICMs after all radiation modalities, 36% of lesions were surgically removed. This contrasts with our review where 25 of 32 cases (78%) after SRS were treated with surgery at some point and only one with repeat SRS (3%). This population is probably biased because asymptomatic patients and those managed conservatively are less likely to be published, leading to an overrepresentation of surgical cases in the literature.

**Table 2:** Case reports of patients treated for cranial nerve schwannoma who later developed RICM.

	Age (y), sex	Dx prompting prior radiation	Location Dx	Type of radiation	Latency	Location RICM	Presenting symptoms	Treatment
Sasagawa et al. (2009)	35, M	VS	CPA	GKRS (12 Gy)	10 y	Pons	Hemihyperesthsia and mild hemiparesis	Conservative
Murakami et al. (2011)	39, W	VS	CPA	GKRS (11.3 Gy)	14 y	CPA	Vestibulocochlear symptoms	Surgery
Murakami et al. (2011)	33, W	TS	Middle cranial fossa	GKRS (12 Gy)	2 y	Middle cranial fossa/prepontine cistern	Trigeminal and abducens symptoms, and contralateral sensorial symptoms	Surgery
Nussbaum et al. (2019)	42, M	VS	CPA	Cyberknife linear accelerator SRS (25 Gy)	11 y	CPA	Facial numbness	Surgery
Graboyes et al. (2025)	78, W	VS	CPA	Fractionated SRT (NR)	16 y	CPA	Hemifacial spasm	Surgery

AVM: Arteriovenous malformation, CM: Cavernous malformation, CN: Cranial nerve, CPA: Cerebellopontine angle, Dx: Diagnosis, GKRS: Gamma knife Surgery, Gy: Grays, M: Male, NR: Not reported, RICM: Radiation-induced cavernous malformation, SRS: Stereotactic surgery, SRT: Stereotactic radiotherapy, VS: Vestibular schwannoma, TS: Trigeminal schwannoma, W: Woman, y: years.

The possibility that the cavernous malformation was sporadic was carefully considered. However, as the lesion developed de novo at advanced age, showed precise spatial correspondence with a previously irradiated volume, and occurred after a latency consistent with known radiation biology. Under these conditions, the likelihood of a coincidental sporadic lesion at that specific location and time is low.

This report has several limitations. First, the RICM was not biopsied; thus, the diagnosis relied on the patient's history of prior radiosurgery in combination with clinical and radiographic findings. Nevertheless, MRI remains the diagnostic modality of choice for cavernous malformations in routine clinical practice, with characteristic features including T2-hyperintense core with surrounding hemosiderin ring creating a “popcorn” appearance (Rigamonti et al., 1987), which mitigates this limitation to some extent. Second, our review includes patients treated with different SRS techniques. Most of the patients were treated in a single session, but some received fractionated treatment. A few were previously treated with conventional fractionated radiotherapy. We acknowledge that combination therapy may have increased the risk of RICM development. Last, the absence of long-term follow-up at the time of this report limits our ability to characterize the lesion's future clinical behavior and validate the conservative management approach.

## 4 Conclusion

RICM represents a rare but clinically significant late complication of radiation therapy that can occur in adults following SRS. This case report highlights the rare occurrence of RICM in the brainstem following GKRS for vestibular schwannoma, adding to the limited body of literature on RICMs associated with SRS. The 19-year latency period represents one of the longest reported for this complication and underscores the importance of long-term follow-up in patients undergoing stereotactic radiation. While often asymptomatic and discovered incidentally, RICMs can cause significant morbidity, including hemorrhage and neurological deficits, requiring individualized management based on symptoms, hemorrhage history, lesion location, and patient factors. No specific guidelines exist for RICM management, but options include observation, surgical resection, or paradoxically, repeat stereotactic radiosurgery in selected cases. Further studies are necessary to clarify the biological mechanisms underlying RICM development, differentiate true cavernomas from radiation-induced pseudocavernomas, establish evidence-based management guidelines, and determine optimal surveillance strategies for patients treated with SRS.

## Ethical statement

The patient gave an informed consent for participating in the case report, documented in her medical journal.

## Funding details

No funding was sought or awarded.

## Declaration of competing interest

The authors report there are no competing interests to declare.
